# Establishing reference intervals for common hematology test parameters from apparently healthy geriatrics in Asella town, Southeast Ethiopia, 2020: a community-based cross-sectional study

**DOI:** 10.3389/fmed.2024.1373283

**Published:** 2024-05-16

**Authors:** Mohammed Hashim Harka, Zuber Siraj, Berhanu Dibaba, Tewodros Zerihun Mamo, Bayyisa Ajema, Aster Tsegaye, Moges Wordofa

**Affiliations:** ^1^Department of Medical Laboratory Science, College of Health Science, Arsi University, Asella, Ethiopia; ^2^Referral Hospital Clinical Laboratory Service, Dilla University, Dilla, Ethiopia; ^3^College of Health Science, School of Medical Laboratory Science, Addis Ababa University, Addis Ababa, Ethiopia

**Keywords:** reference interval, hematological parameters, geriatrics, Southeast Ethiopia, complete blood count

## Abstract

**Background:**

Reference intervals are an important method tool for identifying abnormal laboratory test results. Complete blood count reference values are useful to interpret complete blood count (CBC) results and make clinical decisions, but these values have not been established for geriatrics in Asella town. Therefore, this study aimed to establish reference intervals (RIs) for complete blood count (CBC) parameters from geriatric participants/subjects in Asella town, Southeast Ethiopia.

**Methods:**

A community-based cross-sectional study was conducted from December 2019 to May 2020. An interviewer-administered questionnaire was used to collect data on sociodemography and other characteristics from 342 eligible geriatric participants. Weight, height, and vital signs were measured, and 8 mL of blood sample was collected. Screening tests such as HIV, HBsAg, HCV, syphilis, stool examination, and urinalysis were performed. The hematological parameter was measured using a Sysmex kx-21 hematology analyzer. The data were analyzed using SPSS version 21 software. The non-parametric independent Kruskal–Wallis test and Wilcoxon rank-sum test (Mann–Whitney U test) were used to compare the parameters between age groups and genders. The 97.5 and 2.5th percentile were the upper and lower reference limit for the population.

**Results:**

According to the study’s findings, the reference intervals of red blood cell, white blood cell, platelet count, hemoglobin (HGB), and hematocrit (HCT) in male geriatrics were 3.8–5.85 × 10^12^/L, 3.1–9.66 × 10^9^/L, 115.8–353 × 10^9^/L, 12.4–17.76 g/dL, and 35.06–50.2%, respectively. The respective values for women were 3.94–5.48 × 10^12^/L, 3.13–8.4 × 10^9^/L, 137.5–406 × 10^9^/L, 12.5–16.4 g/dL, and 36.09–48.2%. Most of the hematological parameters showed significant differences between the two genders (*p* value <0.05).

**Conclusion:**

Accurate gender and age-specific reference intervals are crucial in managing patient health. The current study offers essential CBC hematological parameters that can assist clinicians in interpreting laboratory results and can improve healthcare quality in the geriatric population. Therefore, it is more relevant to use the current RIs in the geriatric set-up.

## Introduction

The most important facet of laboratory test interpretation is the concept of reference interval (RI), where test values that fall inside the range are considered normal and those occurring outside the range are considered abnormal (1). The World Health Organization (WHO), the International Federation for Clinical Chemistry (IFCC), and the Clinical Laboratory Standard Institute (CLSI) define reference interval as the group of results obtained by observation or quantitative measurement of an analyte in a selected healthy group of a participant, based on well-defined criteria (2). According to Ceriotti’s definition, reference interval “is an interval that, when applied to the society serviced by the established reference interval, should correctly include most of the subjects with characteristics similar to the reference group and excludes the others” (3).

Moreover, reference interval for many laboratory tests is defined by threshold values between which the test results of a specified percentage (usually 95%) of apparently healthy individuals would fall. The threshold or limiting values for the reference interval are usually 0.025 and 0.975 fractiles of the test result distribution in the reference population. This definition results in the exclusion of the 2.5% of individuals with the lowest results and the 2.5% of individuals with the highest results from the reference interval (4). To establish RI, reference individuals form the reference sample group for measurement of the values from the reference population. Through statistical analysis of the distribution of the obtained values, the reference limits are calculated. These limits then define the reference interval and include them (5).

It has been recommended that RI should be established by selecting a statistically sufficient group (a minimum of 120) of healthy reference subjects. However, it is noted in the guideline that “health is a relative condition lacking a universal definition (6).” However, the selection and recruitment of the appropriate number of reference subjects is difficult, time-consuming, and requires a high price (7). Reference intervals are affected by several factors including age, sex, dietary patterns, altitude, race, and lifestyle among others. For example, hemoglobin concentration, hematocrit, and the other RBC indices reflect the same biological parameters and show similar variability throughout life. Male and female levels were similar in early childhood and increased slowly until 10 years of age before sex differences were observed (8). For that purpose, their determination for every country, even every region, is very crucial.

The determined RI is primarily used for the accurate interpretation of laboratory test results, identifying abnormal and normal results and helping in patient diagnosis and clinical management (9). The hematological test result in a clinical laboratory is a decision-making process, and reference intervals (RIs) produced in the laboratory have an important role in guiding the clinician in interpreting patient results with reference values established from apparently healthy subjects. Therefore, careful establishment or determination of RIs by the laboratory for use in the patient population it serves is important to ensure their proper utility. Most laboratories do not have their own RI for hematological parameters. They are often selected from manufacturer information sheets or outdated publications and may not always be representative of the local population or laboratory setting. However, CBC parameters vary with age and sex, therefore requiring RI which is specific for the society it serves (10). A community-based cross-sectional study conducted in Southwest Ethiopia for the determination of hematological reference interval in the geriatric age group shows that men had higher median and 95% RI for RBC, Hct, and Hgb values than women (11).

Mostly, the reference population widely used includes young adults, and using this may not be appropriate for an elderly patient because there are significant age-related changes observed in hematological parameters (12). Deriving reference values for the older population is particularly problematic. Most laboratories had no specific RI for the geriatric population because this population has a relatively high prevalence of chronic pathologies such as diabetes, dyslipidemia, renal disease, and anemia, and also has comorbidities and regularly takes prescription medications. This makes it difficult to find healthy reference individuals to establish a reference interval (13–16). Reference intervals for African populations are not readily available, and the values used in most African countries including Ethiopia are usually based on the results of measurements in developed countries, which are taken from the literature or package inserts that accompany reagent kits. However, these parameters even in the healthy state are affected by many factors including age, gender, ethnicity, and altitude (17–19). Thus, adopting non-Ethiopian reference values for Ethiopians might be misleading, and adopting adult reference values for geriatrics may impede the detection of pathologies in older populations, so it would be useful to establish age-specific reference values. Given this background, a community-based cross-sectional study was performed to establish common hematological reference values for the geriatrics population in southeast Ethiopia, Oromia regional state, Asella town.

## Materials and methods

### Study setting, study design, and period

A community-based cross-sectional study design was conducted to establish reference intervals for complete blood count parameters from apparently healthy geriatrics in Asella town from December to May 2019. Asella Town is located in the Arsi Zone of the Oromia Region, 126 km (78 mi) south of Addis Ababa.

### Population

The source population for the study consisted of individuals aged 60 years and older, who had been living in the area for at least 6 months. The study population was composed of participants who felt subjectively well and met the eligibility criteria.

### Eligibility criteria

All geriatrics who were aged 60 years and older, feeling subjectively well, free of common bacterial and viral infectious diseases after being screened, and lived for at least 6 months were included. However, geriatrics with known chronic illnesses such as diabetes, hypertension, arthritis, history of TB, history of chronic liver or kidney disease, history of being a hospital inpatient, known history of hematological disorder such as malignancy, and those who are taking pharmacologically active agents, smokers, known carrier state for HBV, HCV, Syphilis, or HIV were excluded.

### Determination of sample size

According to the CLSI recommendation, the sample size was determined using well-defined exclusion and portioning criteria for the selection of the reference individuals. Thus, based on this guideline, the minimum sample size required for RI determination will be 120 healthy individuals for each partitioning (20).

However, according to previous large-scale studies on other African countries, approximately 30% (21) did not meet the inclusion criteria for the establishment of RI for various reasons when screened for common bacterial and viral infectious diseases. Moreover, the minimum sample size will be:


$$N\;\left(100\%-30\%\right)=120$$

$$N\;\left(70\%\right)=120$$

$$N\;\left(0.7\right)=120$$

*N* = 171, this was for one partition, so the value has to be multiplied by 2 which gives 342 for both men and women. The required geriatrics for both genders to achieve a minimum sample size will be 342.

### Sampling method

Five kebeles were selected randomly by the lottery method from eight kebeles in Asella town, and non-probability convenient type sampling techniques were used to recruit all volunteer geriatric individuals from selected kebeles until the sample size was reached. Accordingly, approximately 34 individuals for one partition and 68 for two partitions were selected from each of the five selected kebeles.

### Data collection procedure

Structured and standardized questionnaires were used to collect information on consenting participants to determine the sociodemographic characteristics and health features of study participants. In total, 4 mL of blood samples in ethylenediaminetetraacetic acid (EDTA) vacutainer tubes for full blood count (FBC), Hepatitis B surface antigen (HBsAg), HCV, HIV, and syphilis and 4 mL of blood samples in gel separator for various chemistry tests, stool, and urine samples were also collected and transported to the main laboratory of Arsi University for hematological analysis and screening tests to determine the health characteristics of the study participants. Anthropometric measurements (height and weight) and related physical examination data such as blood pressure were taken on-site. Complete blood count (CBC) and differential blood count will be performed on the blood sample using Sysmex KX-21 N, an automated three-part differential hematology analyzer. Hepatitis B virus was screened using the One Step HBsAg test, and HCV and HIV were screened using the One Step HCV antibody and HIV antibody tests. The interviews and the blood sample collections were performed from 1 January 2020 to 30 April 2020.

### Sample collection procedure

Each participant fasted from food and water for at least 8 h, as common clinical chemistry tests were also determined for this study participant. Blood from each participant was drawn from the cubital vein into appropriate blood collection tubes using vacuum tube needles aseptically. K2EDTA tubes were used for CBC analyses. SST was used for serology and common clinical chemistry tests. Samples collected in SST tubes were separated by centrifugation at 3,000 rpm for 10 min to investigate anti-HCV, anti-HIV, syphilis, and HBsAg levels. All study participants provided urine samples for urinalysis in the red top containers or urine cups and stool samples into the stool cup for stool examination. Samples were transported and tested within 4 h after collection. The stool was examined by wet mount and formal-ether concentration method, and urinalysis was performed using urine dipstick and microscopy.

### Principle of hematological assay

**Figure 1:**
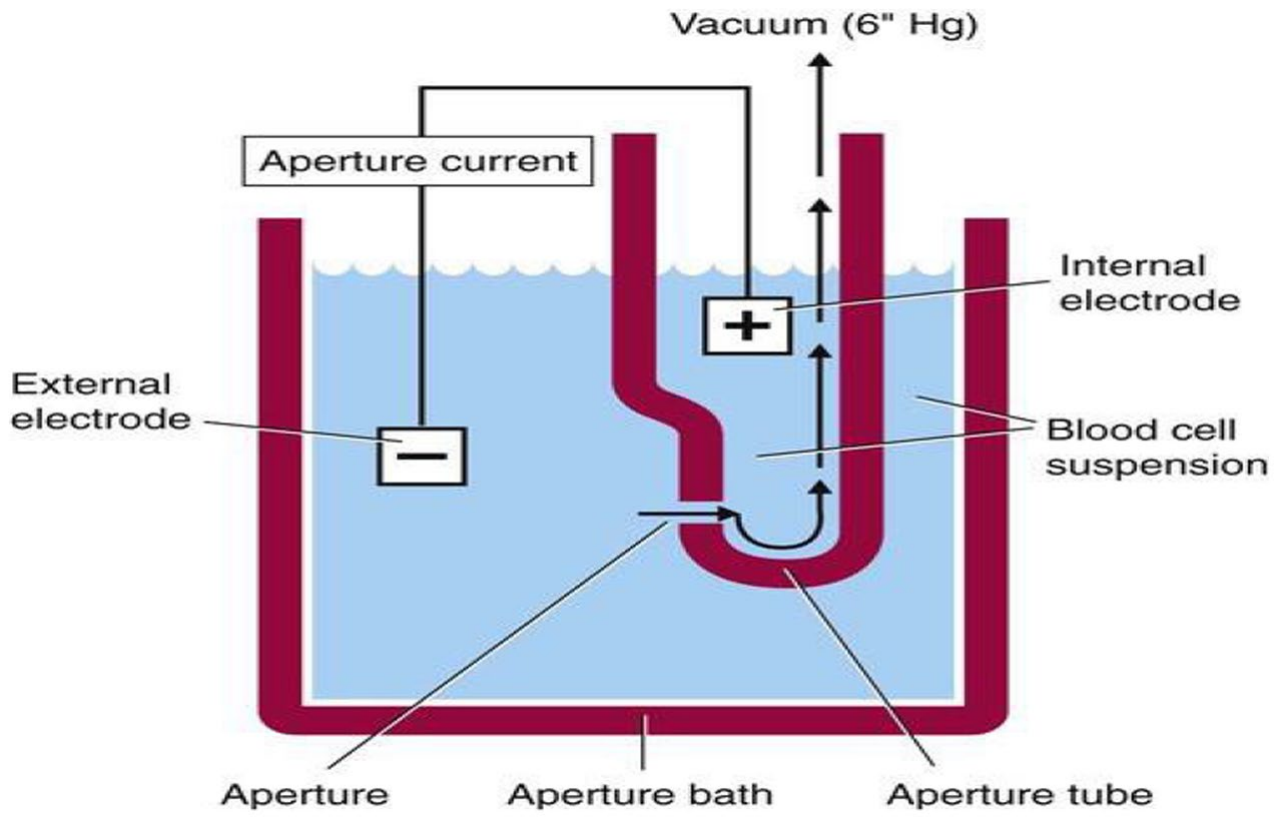
Electrical impedance principle of Sysmex kx-21. WBC: DC detection method. RBC: DC detection method. HGB: Non-cyanide hemoglobin analysis method.

The principle of hematological assay was the impedance or coulter principle, which was based on the detection and measurement of changes in electrical resistance produced by a particle suspended in a conductive liquid as it was drawn through a small aperture. The blood sample is diluted in saline, which is a good conductor of electrical current. DC is applied between the two electrodes. Electrical resistance or impedance occurs as the cells pass through the aperture causing a change in voltage. The change in voltage generates a pulse. Each cell momentarily increases the electrical resistance between two electrodes. The amplitude and size of the pulse depend on the cell volume. A complete blood count (CBC) was performed on the blood sample using Sysmex KX-21 N, an automated three-part differential hematology analyzer. The machine automatically dilutes 50 μL of whole blood sample in the CBC/differential mode, lyses, and directly measures WBC, RBC, HGB, HCT, PLT, LYM #, MIXED #, and NEUT #. The remaining parameters such as MCV, MCH, MCHC, MPV, RDW-CV, and RDW-SD, and differential percentages of LYM%, MIXED%, and NEUT% are calculated or derived from directly measured parameters. The KX-21 N evaluated the counts and sizes of red blood cells (RBC) and platelets (PLT) using electronic resistance detection (Figure 1).

### Variables of the study

#### Dependent variables

The reference interval of common hematological parameters is the dependent variable.

#### Independent variables

In this study, factors that were used as independent variables were age and sex.

### Operational definition

Healthy: An individual who has no signs and symptoms and history of any disease and a negative result for the screening tests.

Geriatric: Individuals of the age group of 60 years and older (≥60).

Hematological parameters: WBC differentials and absolute count, RBC, and platelet parameters.

Reference interval (RI): The 95 percentile interval between the 97.5 and 2.5 percentile which forms the upper and lower reference limit.

### Data quality control

To ensure the quality of the data, a pretest was performed on 5% of the total study population at AURTH to ensure the agreement of the data abstraction format. The data collectors were trained before the data collection process. Supervision and checking were performed to ensure the completeness and consistency of the data. The gathered data were examined for completeness and consistency during data extraction.

### Data analysis procedure

All the data were coded and checked for completeness, then entered into Epidata, and analyzed using SPSS version 21 statistical software for Windows. The data were tested for normality of distribution using the Kolmogorov–Smirnov test; therefore, the non-parametric methods for the determination of RI were used as recommended by CLSI. Median, central 95 percentile, and 90% confidence interval (CI) were calculated. The 97.5 percentile and 2.5 percentile were the upper and lower reference limit for the population. The data for the hematological parameters were collected and analyzed using SPSS version 21 software. The point estimate of the mean and the median with an interval estimate of 2.5 percentile and 97.5 percentile were provided as the reference values. The significant difference between sex groups was determined using the Wilcoxon rank-sum test (Mann–Whitney U test). *p* value <0.05 was considered statistically significant.

## Results

### Sociodemographic characteristics

From 342 geriatrics, 70 individuals (20.5%) were excluded with different exclusion criteria and only 272 geriatrics participated in the study. Among the 272 individuals, 134 (49.3%) were women and 138 (50.7) were men. In total, 36% attained primary education level and 73.2% were orthodox christen followers (Table 1). The mean and age range of the study participants was 65.82 and 60–90 years, respectively. Age categories were grouped by age standardization of a new WHO standard (22).

**Table 1:** Sociodemographic distribution of the study population.

Variable	Sociodemographic characteristics	Frequency	Percentage
Sex	M	138	50.7
	F	134	49.3
	Total	272	100
Age group	60–64	138	50.7
	65–69	64	23.5
	70–74	38	14
	75–79	15	5.5
	≥80	17	6.3
	Total	272	100
Educational status	Illiterate	36	13.2
	Read and Write	37	13.6
	Primary 1–8	98	36
	Secondary 9–12	53	19.5
	College Diploma and above	48	17.6
	Total	272	100
Religion	Catholic	2	0.7
	Protestant	13	4.8
	Muslim	58	21.3
	Orthodox Christian	199	73.2
	Total	272	100
Occupation	Private employee	20	7.4
	Farmer	32	11.8
	Governmental employee	44	16.2
	Housewife	79	29
	Others like pension	97	35.7
	Total	272	100

### Hematological reference intervals

**Figure 2:**
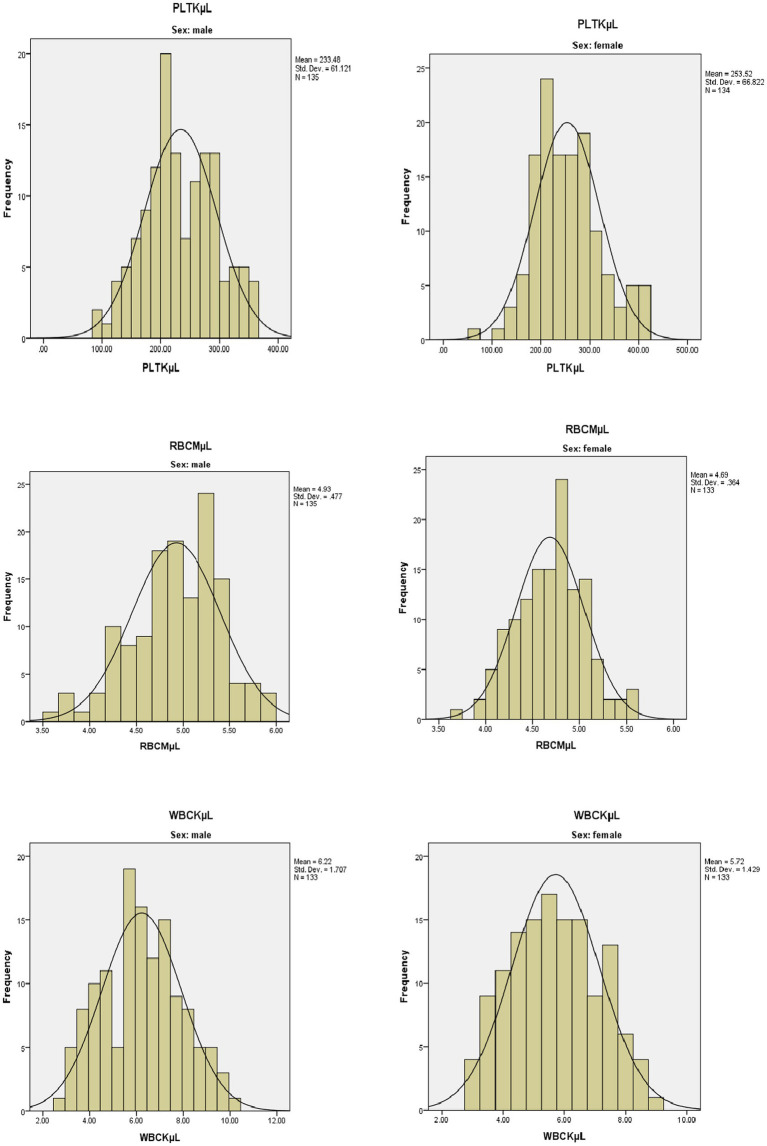
Frequency histogram for selected hematological parameters among men and women in a healthy geriatric population in Asella town, Southeast Ethiopia.

Frequency histograms were prepared to check for normality of some of the hematological parameters and they showed the Gaussian distribution. The following figures showed the Gaussian distribution of the PLT, RBC, and WBC among men and women in the histogram (Figure 2).

The means, median, and reference interval (2.5–97.5 percentile) for male and female geriatric populations are presented in Table 2. The reference intervals were calculated based on the IFCC and the CLSI guidelines. Most of the hematological parameters showed significant differences across genders. Men had higher WBC (3.1–9.66 × 10^9^/L vs. 3.13–8.4 × 10^9^/L), RBC (3.80–5.85 × 10^12^/L vs. 3.94–5.48 × 10^12^), HGB (12.4–17.76 g/dL vs. 12.5–16.4 g/dL), HCT (35.06–50.2% vs. 36.49–48.2%), NEU% (24.06–70.5% vs. 23.67–67.86%), and MIX cell number (0.3–1.56 × 10^9^/L vs. 0.3–1.4 × 10^9^/L) than women, whereas women had higher PLT (137.5–406.0 × 10^9^/L vs. 115.8–353 × 10^9^), and LYM % (21.88–64.79% vs. 18.84–59.2%) than men. On the other hand, the MIX% cells, absolute lymphocyte number, RDWCV, PDW, and MPV did not show statistically significant differences among the genders (*p* > 0.05).

**Table 2:** Mean, medians, and 95% (2.5 and 97.5th percentiles) for the upper and lower limit about sex among health geriatrics in Asella town, Southeast Ethiopia.

Parameter	Sex	Unit	No^*^	Median	Mean	Min	Max	95%		*p* value
								2.5th	97.5th	
WBC	M	10^9^/L	133	6.1	6.22	2.7	10.2	3.1	9.66	0.015
	F		133	5.5	5.71	3	9	3.13	8.4	
RBC	M	10^12^/L	135	5	4.93	3.6	5.95	3.8	5.85	0
	F		133	4.7	4.68	3.69	5.6	3.94	5.48	
HGB	M	g/dL	133	15.5	15.38	11.7	18.3	12.4	17.76	0
	F		130	14.35	14.29	11.4	17.4	12.5	16.4	
HCT	M	%	134	44	43.9	34	50.8	35.06	50.2	0
	F		131	41.3	41.5	34.6	51.3	36.49	48.2	
MCV	M	FL	138	89	89.07	79.4	98.6	81.64	98.15	0.215
	F		131	88	88.49	80.5	98.6	82.39	97.36	
MCH	M	Pg.	138	31.3	31.3	26.2	35.6	27.78	34.8	0
	F		129	30.4	30.5	26.9	34.7	27.25	34.1	
MCHC	M	%	133	35	35.04	33.2	37.1	33.5	36.56	0
	F		127	34.5	34.5	32.6	36.7	32.72	36.26	
PLT	M	10^9^/L	135	224	233	93	364	115.8	353	0.025
	F		134	246.5	253.5	66.8	412	137.5	406	
LYM%	M	%	138	36.1	37.35	11.9	68	18.84	59.2	0.002
	F		133	40.2	41.3	17.8	65.3	21.88	64.79	
							
NUE%	M	%	138	52.5	50.64	20.8	73.6	24.06	70.5	0.02
	F		134	48.6	47.4	16.4	69.9	23.67	67.86	
MIX%	M	%	133	11.6	11.77	1.6	22.2	4.6	21.56	0.23
	F		133	10.4	11.17	4.9	21.9	5.3	21	
LYM NUMBER	M	10^9^/L	134	2.1	2.24	0.6	4	1.14	3.6	0.554
	F		132	2.3	2.28	1.1	4	1.2	3.8	
NUE NUMBER	M	10^9^/L	137	3.2	3.3	0.7	6.9	1.14	6.5	0.004
	F		134	2.85	2.8	0.7	5.5	0.84	5.32	
MIX NUMBER	M	10^9^/L	133	0.7	0.72	0.1	1.7	0.3	1.56	0.003
	F		132	0.6	0.63	0.3	1.5	0.3	1.4	
RDW CV	M	%	133	13.1	13.02	11.1	14.9	11.47	14.66	0.194
	F		129	12.9	12.95	11.4	15.7	11.6	15.6	
PDW	M	Fl	134	12.9	13.2	8.5	18.3	10.33	17.1	0.82
	F		129	13.2	13.23	9.3	18	10.42	17.3	
MPV	M	Fl	137	10.6	10.61	8.7	13.4	8.84	12.67	0.709
	F		133	10.6	10.65	8.5	13.2	9.03	12.96	

^*^The number of study samples shown in the above table was outlier-cleaned data for each parameter.

In the Table 3, the determined RI for MCV, MIX%, LYM NUMBER, RDWCV, PDW, and MPV was combined since they were insignificant among men and women. However, for the rest of the parameters, the RI was established separately because they were significant among the two genders.

**Table 3:** Established reference interval of hematological parameters among healthy geriatrics in Asella town, Southeast Ethiopia.

Parameter	Unit	Sex	Sample number	Lower limit	Upper limit	Lower 90% CI	Upper 90% CI	*p* value
WBC	10^9^/L	M	133	3.1	9.66	2.86–3.34	9.42–9.9	0.015
		F	133	3.13	8.4	2.93–3.33	8.2–8.6	
RBC	1,012/L	M	135	3.8	5.85	3.73–3.87	5.78–5.91	0.000
		F	133	3.94	5.48	3.89–3.99	5.43–5.53	
HGB	g/l	M	133	12.4	17.76	12.2112.58	17.58–17.94	0.000
		F	130	12.5	16.4	12.37–12.63	16.27–16.53	
HCT	%	M	134	35.06	50.2	34.57–35.55	49.71–50.69	0.000
		F	129	36.49	48.2	36.11–36.86	47.82–48.57	
MCV	fL	M/F	269	81.95	97.9	81.56–82.3	97.5–98.3	0.215
MCH	Pg	M	138	27.78	34.8	27.55–28.0	34.57–35.03	0.000
		F	129	27.25	34.1	27.02–27.48	33.87–34.33	
MCHC	Pg	M	133	33.5	36.56	33.38–33.62	36.44–36.68	0.000
		F	127	32.72	36.26	32.6–32.8	36.11–36.14	
PLT	10^9^/L	M	135	115.8	353	107–124	344–361	0.025
		F	134	137.5	406	128–147	396–415	
LYM%	%	M	139	18.84	59.2	17.39–20.3	57.7–60.6	0.002
		F	133	21.88	64.79	20.4–23.4	63.3–66.3	
NEU%	%	M	138	24.06	70.5	22.5–25.7	68.9–72.1	0.02
		F	134	23.67	67.86	22.0–25.3	66.2–69.5	
MIX%	%	M/F	266	5.1	21.2	4.7–5.5	20.8–21.6	0.154
LYM NUMBER	10^9^/L	M/F	266	1.2	3.6	1.1–1.3	3.54–3.66	0.547
NEU NUMBER	10^9^/L	M	137	1.14	6.5	0.9–1.3	6.3–6.7	0.004
		F	134	0.84	5.32	0.68–1.0	5.16–5.48	
MIX NUMBER	10^9^/L	M	133	0.3	1.56	0.26–0.34	1.52–1.6	0.003
		F	132	0.3	1.4	0.26–0.34	1.36–1.44	
RDWCV	%	M/F	262	11.6	14.74	11.56–11.7	14.66–14.82	0.194
PDW	fl	M/F	263	10.4	17.1	10.2–10.6	16.9–17.3	0.8
MPV	fl	M/F	270	8.97	12.9	8.8–9.06	12.8–12.99	0.838

As shown in Table 4, the reference interval was compared to the existing reference interval which is derived from the manufacturer and the manual book of Sysmex kx-21. These values are currently used in our laboratory. The lower limits of the NEU%, absolute NEU count in both women and men, and platelet count in men were comparably lower than the existing RIs. Both the lower and the upper limits showed an increment with the WBC and MCH in men and RBC, MCHC, HGB, HCT, LYM%, LYM Numbers, and slightly mixed count cell values in both men and women when compared to existing reference interval. MCV, PLT, MIX%, and PDW in both women and men and NEU% in women showed a mild decrease in the upper limits as compared to the laboratory RI. In addition, HGB showed the maximum percentage of the study individuals (76% of women and 57% of men) outside the existing reference values, followed by RBC (58.64% of women and 15.5% of men).

**Table 4:** Established reference interval, existing reference interval, and percentage of the outlier of study participants from existing reference interval among healthy geriatric population in Asella town, Southeast Ethiopia.

Parameter	Sex	Number	Established RI (2.5–97.5) percentiles	Existing RI	% of individuals outside the existing RI
WBC	M	133	3.1–9.66	2.6–8.8	6.76
	F	133	3.13–8.4	3.1–10.3	N/A
RBC	M	135	3.8–5.85	3.6–5.3	15.5
	F	133	3.94–5.48	3.2–4.6	58.64
HGB	M	133	12.4–17.76	11.3–15.7	57
	F	130	12.5–16.4	9.9–13.6	76
HCT	M	134	35.06–50.2	32.6–47.5	14.9
	F	131	36.49–48.2	30.2–42.3	38.16
MCV	M	138	81.64–98.15	80.3–103.4	2.17
	F	131	82.36–97.36	78.6–102.2	2.29
MCH	M	138	27.78–34.8	26–34.4	3.6
	F	129	27.25–34.1	25.2–34.7	N/A
MCHC	M	133	33.5–36.56	31.8–36.3	4.5
	F	127	32.72–36.26	31.3–35.4	10.23
PLT	M	135	115.8–353	134–377	4.4
	F	134	137.5–406	128–434	N/A
LYM%	M	138	18.84–59.2	17.5–47.9	17.39
	F	133	21.88–64.79	15–45.8	31.57
MIX%	M	133	4.6–21.56	1.9–24.6	N/A
	F	133	5.3–21.0	1.3–25.9	N/A
NEU%	M	138	24.06–70.5	38.3–69	23.9
	F	134	23.67–67.86	43.7–77.1	33.58
LYM NUMB	M	134	1.14–3.6	0.8–2.7	23.13
	F	132	1.2–3.8	0.9–2.8	16.66
MIX NUMB	M	133	0.3–1.56	0.1–1.5	2.25
	F	132	0.3–1.4	0.1–1.6	N/A
NEU NUMB	M	137	1.14–6.5	1.2–5.3	8
	F	134	0.84–5.32	1.6–6.9	13.43
RDW-CV	M	133	11.47–14.66	10.8–14.9	N/A
	F	129	11.6–15.6	10.6–15.7	N/A
PDW	M	134	10.33–17.1	9.8–18.0	0.74
	F	129	10.42–17.3	9.8–18.0	0.77
MPV	M	137	8.84–12.67	8.1–12.4	2.18
	F	133	9.03–12.96	8.1–12.4	5.26

N/A, Not available.

## Discussion

Reference interval studies are scarce on the elderly in Africa. Existing publications on RI in Africa are generally limited to specific sub-populations: usually children or individuals of ≤60 years. The absence of precise and dependable reference intervals for Africans including the Ethiopian geriatric population has led numerous clinical laboratories to rely on reference values provided by *in vitro* diagnostic company kit inserts, textbooks, or published literature. This practice can potentially lead to erroneous interpretation of laboratory test results, resulting in the misdiagnosis of patients. Recognizing the significance of addressing these crucial problems, this study was conducted to establish reference intervals for hematological complete blood count parameters in healthy geriatric populations in Asella, Southeast Ethiopia. The lower reference limits for RBC and HGB count in this study are lower in male population and higher in female population (RBC 3.8 vs. 4.1 × 10^12^/L in men and 3.94 vs. 3.7 × 10^12^/L in women), (HGB 12.4 vs. 13 in men and 12.5 vs. 11.1 in women) than those reported from the study on complete blood count reference intervals and patterns of changes across pediatric, adult, and geriatric ages in Korea (23).

Gender differences in RBC, HGB, and HCT observed in this study were also similar to studies in six geographical regions in China, a study conducted to establish a reference interval in Canada and population-based hematology reference ranges for older people in rural south-west Uganda, which showed that women had lower values than men (8, 24, 25). This trend was also observed in another study conducted among the healthy Ugandan population, where RBC parameters such as RBC, Hgb, and Hct values showed decrement among women (26). Meanwhile, the much lower HGB levels in women were attributed to decreased metabolic demand, decreased muscle mass, and lower iron stores due to menstruation (27).

This is to compare mean HGB, HCT and RBC between our study with other study. These findings reflected that RBC parameters are affected by altitude. In the current study, the finding was lower when compared to the study in three high-altitude towns, namely, Cojata, Ananea, and Rinconada which are located at greater than 4,355, 4,660, and 5,500 m, respectively, which was higher than our study area which was located at 2,430 m above the sea level (28).

In this study, having a higher mean value of PLT count in women than in men was similar to another study from Chennai, Southern India, which aimed to establish reference intervals for the hematological parameters among individuals aged 18–70 years, which include geriatric population (29). This could be due to a hormonal influence on their regulation. The process by which megakaryocytes proceed to proplatelet formation and platelet production is reportedly under the influence of autocrine estrogen (30). Additionally, estrogen-receptor antagonists inhibit platelet production *in vivo*, supporting the role of estrogens in platelet production (31).

Men have also a higher mixed count and neutrophils than women and appeared to be consistently lower in women than in men across all age groups, which was similar to the study on aging and the hematological profile of the Australian community (12). The difference in mixed count may be due to the presence of allergic and parasitic diseases in the healthy elderly subjects, which is not excluded during screening tests such as parasitic examination or while filling questionnaires, falsely making negative for individuals with allergic conditions (32).

Compared to other reference values established in the Canada Health Measures Survey, the currently established reference interval was lower for the lower limit of PLT (115.8–353 × 10^9^/L) vs. (151.8–324 × 10^9^/L) for men and (137.5–406 × 10^9^/L) vs. (153.2–361 × 10^9^/L) for women and also have both upper and lower limit WBC (3.1–9.66 × 10^9^/L vs. 3.8–10.4 × 10^9^/L) for men and (3.13–8.4 × 10^9^/L vs. 3.8–10.4 × 10^9^/L) for women and NEU number (1.14–6.5 × 10^9^/L vs. 2.0–6.4 × 10^9^/L) for men and both upper and lower limit of NEU number (0.84–5.32 × 10^9^/L vs. 2.0–6.4 × 10^9^/L) for women (8). It was also observed in our study that the complete blood count medians of WBC, RBC, HGB, HCT, and platelet were lower than the study conducted in population-based hematology reference ranges for old people in rural south-west Uganda (25).

The lower and upper limits of the WBC, PLT, and RDWCV in both men and women, the upper limit of RBC, HCT, and HGB in women, the upper and lower limit of HGB and HCT in men, and the upper limit of MCV in both women and men decreased slightly when compared to other reports conducted in southwest Ethiopia. However, the lower limit of RBC, HCT, and HGB in women, lower limit of MCV in both sexes, and lower limit of absolute neutrophil show slight increment (11). This difference in hematological parameters within one country could be due to differences in geographical factors, habits of lifestyle, age group, dietary pattern, and altitude. The overall decrease could be due to the gradual loss of androgens, which stimulate increased production of erythrocytes (33, 34).

Both the upper and lower limits of WBC in both men and women, the upper and lower limits of RBC, HCT, and HGB in men, and the upper limit of HGB and HCT in women in this study were lower when compared to the study established reference intervals for hematology test parameters from apparently healthy geriatric individuals in Southwest Ethiopia (11).

The RI established in this study for WBC, RBC count, Hgb, Hct, MCV, MCH, MCHC, LYM number of mixed cell count, platelet, and RDW differed from the existing reference intervals utilized in Asella Referral and Teaching Hospital, which were taken from the manual book of Sysmex kx-21 (35). The established reference interval for the above parameters was higher than the existing reference interval. This might be due to differences in the study area and setting and the genetics of the study participant. The strength of this study was assessing the disease (acute and chronic) condition of each participant and selecting only eligible participants after performing screening tests for each with challenges such as COVID-19, which makes it difficult while some of the data collected after COVID-19 and processing of laboratory tests.

The study had certain limitations. A fundamental limitation is that the study participants were selected on the basis of their willingness to participate in the study by the convenient sampling method. These factors might create selection bias which may limit the possibility of generalizing the result obtained in this study to the entire geriatrics population in Asella town. Furthermore, it was also not possible to screen for all medical conditions such as inflammation-related disease, drugs, and occupational exposure which may have an influence on the results obtained. Irrespective of the highlighted shortcomings, it is our position that the established RI may have utility for diagnostic laboratories in Asella town. The other limitation is that due to the limitation of resources, RIs for other hematological parameters such as coagulation profiles and ESR were not included in this study.

## Conclusion

Accurate gender and age-specific reference intervals are crucial in managing patient health. The current study offers essential CBC hematological parameters that can assist clinicians in interpreting laboratory results and can improve healthcare quality in the geriatric population. Therefore, it is more relevant to use the current RIs in the geriatric set-up.

## Data availability statement

The raw data supporting the conclusions of this article will be made available by the authors, without undue reservation.

## Ethics statement

The studies involving humans were approved by Departmental Ethical Review and Research Committee (DERC) of Addis Ababa University. The studies were conducted in accordance with the local legislation and institutional requirements. The participants provided their written informed consent to participate in this study.

## Author contributions

MH: Conceptualization, Data curation, Formal analysis, Funding acquisition, Investigation, Methodology, Project administration, Resources, Software, Supervision, Validation, Visualization, Writing – original draft, Writing – review & editing. ZS: Conceptualization, Formal analysis, Investigation, Methodology, Supervision, Visualization, Writing – review & editing. BD: Conceptualization, Formal analysis, Investigation, Methodology, Supervision, Visualization, Writing – review & editing. TZ: Conceptualization, Data curation, Formal analysis, Investigation, Methodology, Supervision, Writing – review & editing. BA: Conceptualization, Data curation, Formal analysis, Investigation, Methodology, Visualization, Writing – review & editing. AT: Conceptualization, Data curation, Formal analysis, Investigation, Methodology, Project administration, Supervision, Validation, Visualization, Writing – review & editing. MW: Conceptualization, Data curation, Formal analysis, Investigation, Methodology, Supervision, Visualization, Writing – review & editing.

## Glossary

CBC: Complete blood count
CD4: Cluster of differentiation 4
CI: Confidence interval
CLSI: Clinical Laboratory Standard Institute
DC: Direct current
DERC: Departmental Ethical Review and Research Committee
EDTA: Ethylene diamine tetra acetic acid
FBC: Full blood count
HBsAg: Hepatitis B surface antigen
HBV: Hepatitis B virus
HCT: Hematocrit
HCV: Hepatitis C virus
Hgb: Hemoglobin
HIV: Human immunodeficiency virus
IFCC: International Federation for Clinical Chemistry and Laboratory Medicine
LYM: Lymphocyte
MCH: Mean corpuscular hemoglobin
MCHC: Mean corpuscular hemoglobin concentration
MCV: Mean cell volume
MPV: Mean platelet volume
MIXED: Basophil, eosinophil, and monocyte
NEUT: Neutrophil
PCV: Packed cell volume
PDW: Platelet distribution width
PLT: Platelet
RBC: Red blood cell
RDW-CV: Red blood cell distribution width coefficient of variation
RDW-SD: Red blood cell distribution width standard deviation
RI: Reference interval
SOP: Standard operating procedure
SST: Serum separator tube
WBC: White blood cell
WHO: World Health Organization

## References

[1] Ridley J. Essentials of Clinical Laboratory Science. Nelson Education (2010).

[2] Wayne PA (2000). Clinical and laboratory standards institute. How to define and determine reference intervals in the clinical laboratory: approved guideline. CLSI Document.

[3] Ceriotti F. Prerequisites for use of common reference intervals. Clin Biochem Rev. (2007) 28:115–21. PMID: 17909616 PMC1994109

[4] Boyd JC. Defining laboratory reference values and decision limits: populations, intervals, and interpretations. Asian J Androl. (2010) 12:83–90. doi: 10.1038/aja.2009.9 20111086 PMC3739683

[5] Solberg HE. Approved recommendation (1987). J Clin Chem Clin Biochem. (1987) 25:645–56. 3612033

[6] Ozarda Y. Reference intervals: current status, recent developments and future considerations. Biochem Med. (2016) 26:5–16. doi: 10.11613/BM.2016.001, PMID: 26981015 PMC4783089

[7] Solberg HE. Approved recommendation (1986) on the theory of reference values. Part 1. The concept of reference values. Clin Chim Acta. (1987) 165:111–8. doi: 10.1016/0009-8981(87)90224-5, PMID: 3608186

[8] Adeli K Raizman JE Chen Y Higgins V Nieuwesteeg M Abdelhaleem M. Complex biological profile of hematologic markers across pediatric, adult, and geriatric ages: establishment of robust pediatric and adult reference intervals on the basis of the Canadian health measures survey. Clin Chem. (2015) 61:1075–86. doi: 10.1373/clinchem.2015.240531, PMID: 26044509

[9] Zeh CE Odhiambo CO Mills LA. Laboratory reference intervals in Africa. Blood Cell. (2012) 15:303–20. doi: 10.5772/48250

[10] Ambayya A Su AT Osman NH Nik-Samsudin NR Khalid K Chang KM. Haematological reference intervals in a multiethnic population. PLoS One. (2014) 9:e91968. doi: 10.1371/journal.pone.0091968, PMID: 24642526 PMC3958408

[11] Bimerew LG Demie T Eskinder K Getachew A Bekele S Cheneke W. Reference intervals for hematology test parameters from apparently healthy individuals in Southwest Ethiopia. SAGE Open Med. (2018) 6:205031211880762. doi: 10.1177/2050312118807626 PMC6207960 30397473

[12] Brightwell RF Crawford GPM Cale JB Pedler PJ Bittles AH. Ageing and the haematological profiles of an Australian community. Ann Hum Biol. (1998) 25:1–10. doi: 10.1080/03014469800005382, PMID: 9483203

[13] Mitchell-Fearon K Waldron N James K Laws H Holder-Nevins D Eldemire-Shearer D. Hypertension and diabetes prevalence in older persons in Jamaica, 2012. West Ind Med J. (2014) 63:416–23. doi: 10.7727/wimj.2014.065, PMID: 25781276 PMC4655690

[14] Patel KV. Epidemiology of anemia in older adults. In: Seminars in hematology. Philadelphia, Pennsylvania, United States: WB Saunders. (2008) 45:210–217. 18809090 10.1053/j.seminhematol.2008.06.006 PMC2572827

[15] Marengoni A Winblad B Karp A Fratiglioni L. Prevalence of chronic diseases and multimorbidity among the elderly population in Sweden. Am J Public Health. (2008) 98:1198–200. doi: 10.2105/AJPH.2007.121137, PMID: 18511722 PMC2424077

[16] Nobili A Franchi C Pasina L Tettamanti M Baviera M Monesi L. Drug utilization and polypharmacy in an Italian elderly population: the EPIFARM-elderly project. Pharmacoepidemiol Drug Saf. (2011) 20:488–96. doi: 10.1002/pds.2108, PMID: 21264988

[17] Nduka N Aneke C Maxwell-Owhochuku S. Comparison of some haematological indices of Africans and Caucasians resident in the same Nigerian environment. Haematologia. (1988) 21:57–63. PMID: 3396976

[18] Horn PS Pesce AJ. Effect of ethnicity on reference intervals. Clin Chem. (2002) 48:1802–4. doi: 10.1093/clinchem/48.10.1802 12324505

[19] El-Hazmi MAF Warsy AS. Normal reference values for hematological parameters, red cell indices, HB A2 and HB F from early childhood through adolescence in Saudis. Ann Saudi Med. (2001) 21:165–9. doi: 10.5144/0256-4947.2001.165, PMID: 17264543

[20] Gary LH Sousan A Boyd JC Ceriotti F Garg U Horn P. Defining, Establishing, and Verifying Reference Intervals in the Clinical Laboratory: Approved Guideline. 3rd ed. Pittsburgh, PA: Clinical and Laboratory Standards Institute (2010).

[21] Stevens W Kamali A Karita E Anzala O Sanders EJ Jaoko W. Baseline morbidity in 2,990 adult African volunteers recruited to characterize laboratory reference intervals for future HIV vaccine clinical trials. PLoS One. (2008) 3:e2043. doi: 10.1371/journal.pone.0002043, PMID: 18446196 PMC2312327

[22] Ahmad OB Boschi-Pinto C Lopez AD Murray CJL Lozano R Inoue M (2001). Age standardization of rates: a new WHO standard. World Health Organization, Geneva. p. 9.

[23] Nah E-H Kim S Cho S Cho H-I. Complete blood count reference intervals and patterns of changes across pediatric, adult, and geriatric ages in Korea. Ann Lab Med. (2018) 38:503–11. doi: 10.3343/alm.2018.38.6.503, PMID: 30027692 PMC6056383

[24] Wu X Zhao M Pan B Zhang J Peng M Wang L. Complete blood count reference intervals for healthy Han Chinese adults. PLoS One. (2015) 10:5–10. doi: 10.1371/journal.pone.0119669 PMC4358890 25769040

[25] Mugisha JO Seeley J Kuper H. Population based haematology reference ranges for old people in rural south-West Uganda. BMC Res Notes. (2016) 9:1–9. doi: 10.1186/s13104-016-2217-x 27604101 PMC5013643

[26] Lugada ES Mermin J Kaharuza F Ulvestad E Were W Langeland N. Population-based hematologic and immunologic reference values for a healthy Ugandan population. Clin Diagn Lab Immunol. (2004) 11:29–34. doi: 10.1128/CDLI.11.1.29-34.2004, PMID: 14715541 PMC321349

[27] Wakeman L Al-Ismail S Benton A Beddall A Gibbs A Hartnell S. Robust, routine haematology reference ranges for healthy adults. Int J Lab Hematol. (2007) 29:279–83. doi: 10.1111/j.1365-2257.2006.00883.x 17617078

[28] León-Velarde F Gamboa A Chuquiza JA Esteba WA Rivera-Chira M Monge CC. Hematological parameters in high altitude residents living at 4355, 4660, and 5500 meters above sea level. High Alt Med Biol. (2000) 1:97–104. doi: 10.1089/15270290050074233, PMID: 11256567

[29] Subhashree AR Parameaswari PJ Shanthi B Revathy C Parijatham BO. The reference intervals for the haematological parameters in healthy adult population of Chennai, southern India. J Clin Diagn Res. (2012) 6:1675. doi: 10.7860/JCDR/2012/4882.2630 23373026 PMC3552202

[30] Nagata Y Yoshikawa J Hashimoto A Yamamoto M Payne AH Todokoro K. Proplatelet formation of megakaryocytes is triggered by autocrine-synthesized estradiol. Genes Dev. (2003) 17:2864–9. doi: 10.1101/gad.1128003, PMID: 14665668 PMC289146

[31] Segal JB Moliterno AR. Platelet counts differ by sex, ethnicity, and age in the United States. Ann Epidemiol. (2006) 16:123–30. doi: 10.1016/j.annepidem.2005.06.052, PMID: 16246584

[32] Adetifa IMO Hill PC Jeffries DJ Jackson-Sillah D Ibanga HB Bah G. Haematological values from a Gambian cohort–possible reference range for a west African population. Int J Lab Hematol. (2009) 31:615–22. doi: 10.1111/j.1751-553X.2008.01087.x 18631172

[33] Cui YG Tong JS Pan QQ Di FS Jia Y Feng T. Effect of androgen on erythropoietin in patients with hypogonadism. Nat J Androl. (2003) 9:248–51. 12931361

[34] Paul AK Latif ZA Iqbal S Amin F Shefin SM Ashrafuzzaman SM. Androgen versus erythropoietin for the treatment of anaemia of pre-dialysis chronic kidney disease. MMJ. (2012) 21:125–8. PMID: 22314467

[35] Corporation Operator’s Manualautomated Hematology ANALYZERKX-21 (2000). Japan: Sysmex KX-21 Operator’s manual—revised October 1998; 306.

